# Detection and analysis of tick-borne infections in communal dogs of northwest Zimbabwe

**DOI:** 10.4102/jsava.v92i0.2096

**Published:** 2021-04-29

**Authors:** Melissa A. Kennedy, Riley E. Thompson, Anna McRee Bakker, Canny Fung, Jessica Dawson, Roger Parry, Chris Foggin, Agricola Odoi

**Affiliations:** 1 Department of Biomedical and Diagnostic Sciences, College of Veterinary Medicine, University of Tennessee, Knoxville, Tennessee, United States of America; 2 Department of Clinical Sciences, Colorado State University, Fort Collins, Colorado, United States of America; 3 Nashville Zoo at Grassmere, Nashville, Tennessee, United States of America; 4 All Pets Health Centre, Spring Hill, Tennessee, United States of America; 5 Long Island Veterinary Specialist, Plainview, New York, United States of America; 6 Victoria Falls Wildlife Trust, Victoria Falls, Zimbabwe

**Keywords:** canine, tick, Southern Africa, *Ehrlichia*, one health

## Abstract

Domestic dogs (*Canis familiaris*) may serve as a reservoir or a sentinel for infectious disease pathogens that can affect human and wildlife health. To understand the role of tick-borne diseases in rural and lesser developed regions, we investigated the prevalence of several tick-borne pathogens in communal dogs of Zimbabwe. Blood samples from 225 dogs in northwest Zimbabwe were assessed by serology for *Ehrlichia canis, Anaplasma phagocytophilum* and *Borrelia burgdorferi*, and 241 samples were assessed by polymerase chain reaction (PCR) for *Ehrlichia*. There was a high seroprevalence (73%) of *E. canis*-specific antibodies in domestic dogs in northwest Zimbabwe, but follow up analyses via PCR and genetic sequencing indicated only 7.5% of the canines were actively infected with the organism. Whilst indicating that an organism serologically related to *E. canis* is likely present in the region, this data also shows that the organism is currently present in a relative minority of the domestic dogs in the region. Its presence as evidenced by both serologic and PCR analysis is significant because of the ‘one health’ paradigm, where humans and wildlife may be affected by the exposure to this pathogen in domestic dogs.

## Introduction

Tick-borne diseases pose a significant risk to humans and domestic animals as well as wildlife in sub-Saharan Africa (Dantas-Torres, Chomel & Otranto 2012; Lightfoot & Norval 1981; Perry & Young 1995). Dogs may be the reservoirs of several infectious agents of significance, including tick-borne pathogens, and can serve as sentinels indicating when specific pathogens may be present in an area (Cleaveland et al. 2006; Day 2011; McRee et al. 2014). These agents directly impact the health and welfare of domestic dogs and may also have significant effects on wildlife and humans as well as other domestic species with which they interact (Cleaveland et al. 2006). Specifically, the ‘One Health’ paradigm, which recognises the interconnectivity of humans, animals and the environment, has been used to demonstrate that domestic dogs and cats are involved in transmission cycles of some tick-borne diseases (Skotarczak 2018). Likewise, endangered African wild dogs (*Lycaon pictus*) show increased pathogen exposure when in contact with domestic dogs (Woodroffe et al. 2012).

Ticks bites can transmit the bacterial pathogens *Ehrlichia* spp., *Anaplasma* spp. and *Borrelia burdorferi* (Groves et al. 1975; Johnson et al. 1984; Stuen 2007). Canine ehrlichiosis is known to occur in South Africa with seroprevalence levels of up to 75% with a higher prevalence in economically depressed areas, yet the causative organism has neither been isolated nor characterised (Inokuma et al. 2005; Pretorius & Kelly 1998). There is even less definitive data from other parts of Africa. A serological survey for antibodies reactive with *E. canis* done on 228 dogs in rural Zimbabwe in 2004 showed evidence of exposure in over a third of the dogs tested (Kelly, Eoghain & Raoult 2004). Only one of these pathogens, *B. burdorferi* (Lyme disease), can be prevented by vaccination, and Lyme disease has not yet been isolated in Zimbabwe. Furthermore, many people in rural Zimbabwe lack the financial resources to purchase vaccines for their dogs. Additionally, tick control is important for prevention of these tick-borne bacterial pathogens, although medications available for tick control in dogs are also expensive.

Good quality data on the importance and means of control of infectious diseases in sub-Saharan Africa are deficient partly because of lack of access to veterinary attention as well as economic barriers (McDermott & Arimi 2002). Since many infectious pathogens affect multiple hosts, it is often more feasible, less expensive and less risky to determine the prevalence of various infectious agents in a domestic species – one that shares an environment with human and wildlife populations. Our objective was to investigate the seroprevalence of three bacterial agents carried by ticks (*Ehrlichia canis, Anaplasma phagocytophilum* and *Borrelia burgdorferi*). In addition, detection of the most seroprevalent infectious agent related to *E. canis* was undertaken using PCR on whole blood.

## Research methods and design

### Sites

Dogs inhabiting the Hwange District Rural communal lands bordering both the Victoria Falls and the Zambezi National Park were used for this investigation. These lands are in the vicinity of wildlife preserves and encompass a major township in Zimbabwe. The annual rainfall in the Hwange District is less than 450 mm annually, and the temperatures are as high as 25 °C and above (Nhemachena et al. 2014). Sampling was performed during periodic cattle dipping at 13 established sites: six in 2012 (Breakfast, Chidobe, Chizuma, Donrovan, Kachechete and Woodland) and seven in 2014 (Chidobe, Chinotimba, Lupinyu, Mflandawonye, Monde, Mosi-Oa-Tunya and Mvuti). Chidobe was the only site visited both years.

### Sampling

Dogs were aged based on appearance, dentition and history when known. Dogs were classified as adults (> 3 years old), young adults (1–3 years old) and juvenile (< 1 year). Dog owners were encouraged to participate in the study by providing donated multivalent canine distemper-canine adenovirus-2-canine parvovirus-2 and rabies vaccines (Merial, Atlanta, GA, United States [US]) as incentives.

### Enzyme-linked immunosorbent assay

Serum samples (*n* = 225) collected in 2012 were tested for antibodies to *E. canis, A. phagocytophilum, B. burgdorferi* and *Dirofilaria immitis* using the IDEXX 4DX SNAP enzyme-linked immunosorbent assay (ELISA) (IDEXX, Inc., Portland, ME, US) following manufacturer instructions. *Borrelia burgdorferi* was primarily investigated because of its inclusion in the ELISA kit; however, evaluating potential spread of Lyme disease was a secondary benefit. Serologic testing on samples collected in 2014 was not done, as kits were unavailable.

### Polymerase chain reaction (PCR) and genetic analysis

To detect *Ehrlichia* organisms, we first extracted whole blood using the QIAamp deoxyribonucleic acid (DNA) Blood Mini Kit (Qiagen, Valencia, CA, US) in Zimbabwe. Extracted DNA was then transported at room temperature to the University of Tennessee College of Veterinary Medicine laboratory and stored at −80 °C until amplification of an ~345 base pair (bp) fragment of the 16S recombinant DNA (rDNA) of *Ehrlichia* (Branger, Rolain & Raoult 2004). A total of 241 canine blood samples were screened for *Ehrlichia* with this method (120 from 2012 and 121 from 2014). Rather than incorporating SYBR green (a Synergy Brand Inc. nucleic acid dye used in quantitative PCR) into the reaction mixture, PCR was performed with conventional methods and amplified products were visualised by agarose gel electrophoresis and gel staining. Amplified products were purified using the QIAquick Gel Extraction Kit (Qiagen, Valencia, CA, US) per manufacturer instructions and genetically sequenced at the Molecular Biology Facilities of the University of Tennessee (Knoxville, TN, US). Resulting sequences were analysed using Lasergene 9 (DNASTAR, Inc., Madison, WI, US) and compared to known sequences in GenBank (Basic Local Alignment Search Tool [BLAST], National Center for Biotechnology Information [NCBI]).

### Data analysis

Serological and PCR results were classified and recorded as positive or negative for each pathogen tested. Crude and factor-specific seroprevalence of each pathogen was then computed. Factors considered were animal age, sex and geographic location. Associations between seroprevalence and each of the factors were assessed using Fisher’s exact test. A *p*-value of < 0.05 was used to indicate statistical significance.

## Results

### Demographic data

Dogs tested in 2012 were primarily (84%) young adults. There were more males (68%) than females (32%), and more samples were tested in July (71%) than in June (29%) 2012. In 2014, 90% of the samples were collected from young adult dogs, and all samples were collected in mid-July.

### Serologic data

Based on serologic testing 10%, 0%, 1% and 73% of the dogs had been exposed to *Anaplasma, Borrelia, Dirofilaria* and *Ehrlichia*, respectively. There was no association between seroprevalence of *Ehrlichia* with either age (*p* = 0.4435) or sex (*p* = 0.0945). However, there was a significant (*p* = 0.0029) association between location and seroprevalence of *Ehrlichia*, with Chisuma having the highest seroprevalence (86.7%; 39/45), followed by Woodland (76.9%; 30/39), Breakfast (75.9%; 22/29), Donrovan (71.1%; 27/38), Kachechete (70.3%; 26/37) and Chidobe (35%; 7/20). Also, there was a significantly (*p* = 0.0051) higher seroprevalence of *Ehrlichia* in July (78%; 118/151) than in June (57%; 33/57).

### Polymerase chain reaction data

A total of 241 (120 from 2012 and 121 from 2014) samples were tested for *Ehrlichia* using PCR. The overall prevalence of *Ehrlichia* based on the PCR results was 7.5% (18/241), all of which were most homologous by genetic sequencing to *E. canis.* Thus, this organism was determined to be an E. canis-like organism. There was a significant (*p* = 0.044) association between the organisms’ prevalence based on PCR test and year with 2012 having a lower prevalence (4.17%; 5/120) than 2014 (10.74%; 13/121). There were no significant (*p* = 0.592) sex differences in the prevalence of *Ehrlichial canis*-related organism during 2014 based on PCR results. However, there was a significant (*p* < 0.001) association between location and its prevalence based on PCR results in 2014 with Lupinyu having the highest prevalence (80%; 4/5), followed by Mvuti (24%; 6/25), Mosi-Oa-Tunya (5.41%; 2/37) and Chidobe (4%; 1/25). The remaining three locations had no positive samples.

## Discussion

This investigation shows a significant level of exposure to an *E. canis*-related organism amongst communal dogs in Zimbabwe. For those whom serologic assays alone were done, it is unknown when exposure occurred. Genetic detection of the organism revealed a much lower prevalence level for active current infection. Testing was done opportunistically during June and July of 2012 and 2014. This corresponds to winter, or the dry season in Zimbabwe, when tick populations are not expected to be at their highest. Nevertheless, the organism was detected and found to be closely related to *E. canis*. The majority of dogs tested were young adults; even so, there was not an age association with exposure. That is, exposure likely occurs very early in a dog’s life, as soon as the first tick season after birth. There was not an even distribution of exposed dogs in the region tested, but exposure was found at a considerable level in all areas.

Because there is extensive serologic cross reactivity amongst ehrlichial species, we cannot say for certain that other species of ehrlichial organisms such as *E. chaffeensis* and *E. ruminantium* or even uncharacterised organisms are not present (Kelly et al. 2004). Whilst all PCR-positive results here identified infections because of *E. canis*, it is possible that other ehrlichial organisms may be present in dog populations. It is unclear as to which tick species is associated with these pathogens. Further studies of ticks involved are needed in order to determine vector associations.

Many tick-borne diseases are endemic in Africa, including organisms related to *Ehrlichia canis* and *Anaplasma phagocytophilum*. A growing number of new *Ehrlichia* organisms are being recognised (Allsopp & Allsopp 2001) that may affect dogs. Unfortunately, in Zimbabwe, *Ehrlichia* and *Anaplasma* have not yet been directly isolated from tick species to determine the vector; however, a recent survey in South Africa determined that *E. canis* was identified in *Rhipicephalus sanguineus* ticks, and that both *Rhipicephalus sanguineus* and *Haemaphysalis elliptica* species carry *A. phagocytophilum*, but not the *Amblyomma* ticks (Mtshali et al. 2017). Related organisms that infect humans are also carried by these vectors and occur in Africa, including *E. chaffeensis* and *E. ewingii* (Brouqui et al. 1994; Ndip et al. 2005). In 2000, an organism related to *A. phagocytophilum*, the agent of human and canine granulocytic anaplasmosis was identified in dogs in South Africa (Inokuma et al. 2005). The significance of all these infectious agents amongst animals in Zimbabwe remains unknown. There is a critical need to determine the prevalence of both *Ehrlichia* and *Anaplasma* in the domesticated canine population to identify the potential vectors associated with those canines and to characterise the risk factors associated with each pathogen. Without this knowledge, it will not be possible to manage these ticks and minimise pathogen transmission of these emerging and preventable diseases.

In summary, testing of communal dogs in Zimbabwe revealed a high seroprevalence for organisms related to *E. canis*. Polymerase chain reaction detection of the organism indicated that there is significant identity with *E. canis*, but as only 18 samples produced positive results, it is possible other serologically related organisms may also be involved.
